# Project DECIDE, part 1: increasing the amount of valid advance directives in people with Alzheimer’s disease by offering advance care planning—a prospective double-arm intervention study

**DOI:** 10.1186/s12910-022-00854-0

**Published:** 2022-12-09

**Authors:** Stefanie Baisch, Christina Abele, Anna Theile-Schürholz, Irene Schmidtmann, Frank Oswald, Tarik Karakaya, Tanja Müller, Janina Florack, Daniel Garmann, Jonas Karneboge, Gregor Lindl, Nathalie Pfeiffer, Aoife Poth, Bogdan Alin Caba, Martin Grond, Ingmar Hornke, David Prvulovic, Andreas Reif, Heiko Ullrich, Julia Haberstroh

**Affiliations:** 1 Faculty V: School of Life Sciences, Institute of Psychology, Psychological Aging Research (PAR), University of Siegen, Adolf-Reichwein-Str. 2a, 57068 Siegen, Germany; 2 University Medical Center of the Johannes Gutenberg University, Mainz, Germany; 3 Goethe University Frankfurt, Frankfurt am Main, Germany; 4 University Hospital Frankfurt, Frankfurt am Main, Germany; 5 Kreisklinikum Siegen, Siegen, Germany; 6 Würdezentrum, Frankfurt am Main, Germany

**Keywords:** Dementia, Alzheimer’s disease, Memory clinic, Advance directive, Patient empowerment, Advance care planning, Autonomy, Supported decision making

## Abstract

**Background:**

Everybody has the right to decide whether to receive specific medical treatment or not and to provide their free, prior and informed consent to do so. As dementia progresses, people with Alzheimer’s dementia (PwAD) can lose their capacity to provide informed consent to complex medical treatment. When the capacity to consent is lost, the autonomy of the affected person can only be guaranteed when an interpretable and valid advance directive exists. Advance directives are not yet common in Germany, and their validity is often questionable. Once the dementia diagnosis has been made, it is assumed to be too late to write an advance directive. One approach used to support the completion of advance directives is ‘Respecting Choices’^®^—an internationally recognised, evidence-based model of Advance Care Planning (ACP), which, until now, has not been evaluated for the target group of PwAD. This study’s aims include (a) to investigate the proportion of valid advance directives in a memory clinic population of persons with suspected AD, (b) to determine the predictors of valid advance directives, and (c) to examine whether the offer of ACP can increase the proportion of valid advance directives in PwAD.

**Method:**

We intend to recruit at least *N* = 250 participants from two memory clinics in 50 consecutive weeks. Of these, the first 25 weeks constitute the baseline phase (no offer of ACP), the following 25 weeks constitute the intervention phase (offer of ACP). The existence and validity of an advance directive will be assessed twice (before and after the memory clinic appointment). Moreover, potential predictors of valid advance directives are assessed.

**Discussion:**

The results of this study will enhance the development of consent procedures for advance directives of PwAD based on the ACP/Respecting Choices (R) approach. Therefore, this project contributes towards increasing the autonomy and inclusion of PwAD and the widespread acceptance of valid advance directives in PwAD.

*Trial Registration* DRKS, DRKS00026691, registered 15th of October 2021, https://www.drks.de/drks_web/navigate.do?navigationId=trial.HTML&TRIAL_ID=DRKS00026691

## Background

The right to make autonomous decisions is enshrined in law. Individual autonomy encompasses self-determined decision-making in medical contexts. PwAD have (like every other person) the right to decide whether to receive a specific medical treatment or not and to provide their free, prior, and informed consent to this treatment. To make treatment decisions, PwAD need to participate in an informed consent process, which requires that: (1) a competent person, (2) makes a free choice, and (3) following adequate information disclosure [1]. However, as dementia progresses, PwAD can lose their capacity to provide informed consent to complex medical treatment because of deterioration in cognitive function.

Despite such loss of capacity to consent, the availability of an interpretable and valid advance directive, an instrument to express the will of the affected person, can be considered to preserve their autonomy [2, 3]. To ensure the validity of an advance directive, its preparation presupposes informed consent that fulfils the above-mentioned three criteria. Important elements of informed consent are comprehension of one’s diagnosis, the progress of the illness and existing treatment options, and existing options at the end of life [4].

The use of advance directives is still not sufficiently widespread in Germany (approx. 51% of people above 60 years, [5]), and the interpretability and validity of those that do exist are questionable in about 50% of cases (e.g., regarding the question: Was the person capable of consent when preparing the advance directive?) [6]. The proportion of questionable cases is presumed higher in PwAD. Once the dementia diagnosis has been made, it is assumed to be too late to prepare an advance directive because the capacity to complete an advance directive (legal term: the capacity to consent) is considered questionable even when dementia is mild. In an older study, only one-fifth of those with early dementia (mostly of higher average premorbid intelligence) were considered competent to complete an advance directive [7].

The UN Convention on the Rights of Persons with Disabilities (UN-CRPD, ratified by Germany in 2009) is a human rights treaty that grants persons with disabilities, such as PwAD, the freedom to make their own choices (UN-CRPD, Article 3(1)). Therefore, according to the law, PwAD are considered as persons with valid legal capacity (UN-CRPD, Article 12). State parties are obliged to support their ability to make legally valid decisions.

One approach used to support the completion of advance directives is ‘Respecting Choices’®—an internationally recognised, evidence-based model of ACP, which, until now, has not been evaluated for the target group of PwAD. In Germany, according to § 43 Social Security Code XI, financing for ACP is only available to people living in nursing homes. Comparable services do not yet exist for those that have not been institutionalised.

### Study aims, research questions & hypotheses

The study’s aims include (a) to investigate the proportion of valid advance directives in a memory clinic population of suspected PwAD, (b) to determine the predictors of valid advance directives, and (c) to examine whether the offer of ACP can increase the proportion of valid advance directives in PwAD.

This study consists of two parts:

In part 1, we assess the status quo of advance directives in memory clinics. Part 1 is descriptive and explorative and uses an observational single-group design to answer the following research questions:

1. What is the proportion of patients with (valid) advance directives in the population of memory clinic patients?
2. How satisfied are memory clinic patients with their advance directives?
3. Do demographic variables (age, gender, education, health literacy, need for autonomy, comorbidities, and/or dementia status) predict the existence and validity of advance directives?
  Study part 2 investigates the effects of offering ACP to memory clinic patients and is conceived as a non-randomized comparative trial with a baseline and an intervention phase. It is assumed that the offer of ACP is superior to no intervention (“care as usual”). The following hypotheses are investigated:
4. When offered to take part in ACP (the intervention phase of the study), the proportion of patients with (valid) advance directives will be significantly higher than in the preceding baseline phase without the offer of ACP.
5. When offered to take part in ACP (intervention phase of the study), the satisfaction of patients with their advance directives will be significantly higher than in the preceding baseline phase without the offer of ACP.

This study is a part of the comprehensive research project DECIDE (*Deci*sion-making places in Alzheimer’s *de*mentia—supporting advance decision-making by improving person-environment fit). DECIDE aims to enable PwAD benefit from their right to self-determination as much as possible.

## Methods

### Settings and participants

Participants will be recruited from two outpatient memory clinics (University Hospital Frankfurt, Germany; Kreisklinikum Siegen, Germany). We plan to recruit at least *n* = 125 participants at each location, with a total of *N* = 250 participants. This will be a convenience sample: Each patient presenting in either of the two clinics during the data assessment period (25 weeks for baseline and intervention phase, respectively) will be asked for participation as long as the inclusion criteria are fulfilled.

Patients are eligible for participation in the study if they present with suspected (mild or moderate) dementia. Since the capacity to consent to study participation could be an issue, the inclusion process will be based on the decision tree for the inclusion of non-consenting individuals in medical research [8]. Also, this allows us to include participants with questionable capacity to consent.

Exclusion criteria for study part 1 (status quo) are lack of capacity to consent to medical research with simultaneous incapacity for supported decision making by a relative or proxy or the lack of participant’s assent.

Exclusion criteria for study part 2 (intervention: ACP offer) are a final diagnosis of severe dementia by clinical rating, delirium, intellectual disability, severe mental illness, lack of capacity to consent to medical research with simultaneous incapacity for supported decision making by a relative or proxy, no assent by the patient, uncompensated pronounced sensory deficits, or insufficient knowledge of the German language, which makes the understanding of the study documents and/or the interview impossible.

In both study parts, there are no restrictions regarding concomitant interventions. Simulations were performed to determine the power of the analysis for our principal hypothesis: The proportion of valid advance directives increases when patients are offered ACP. Assuming that the proportion of valid advance directives during the baseline phase is 25% and can increase to 50% by offering of ACP and that an average of five patients are included weekly, the power is approximately 95%. We are positive to reach this number of participants; nonetheless, the sample size is constrained by the capabilities of the two participating outpatient memory clinics.

### Study design, intervention, and outcomes

In study part 1 (status quo), the overall sample is used in an observational cross-sectional single-group design. Outcomes are (1) the presence of an advance directive and its validity, (2) the patients’ satisfaction with this advance directive regardless of its validity, all assessed when patients present at the memory clinic for the first time.

In addition to this descriptive part, potential predictors of the presence of a (valid) advance directive are investigated. Variables of interest are demographic variables (e.g., age, gender, education), health literacy, need for autonomy in medical decision-making, cognitive status measured by the Mini-Mental State Examination (MMSE), mental health determined by the Geriatric Depression Scale (GDS), and physical and mental health status measured by the Somatic Morbidity Index (SMI) or the mental health score, respectively, of the Cumulative Illness Rating Scale—Geriatric (CIRS-G).

Study part 2 (intervention) uses a two-sample design with a blockwise allocation to baseline and intervention group. Hence, there is a baseline phase of 25 weeks, during which none of the participants receives ACP, and an intervention phase of 25 weeks, during which every participant receives ACP.

The intervention is the *offer* of an ACP (no: baseline phase/yes: intervention phase), but not the ACP process itself. The offer is made by the treating physician at the memory clinic, who also performs the informed consent procedure and collects the data.

The outcomes in this study part are the same as in study part 1: (1) the presence of an advance directive and its validity, and (2) the patients’ satisfaction with the existing advance directive (valid or not); always referring to the participant’s latest advance directive.

### Procedure

#### Data collection

**Fig. 1:**
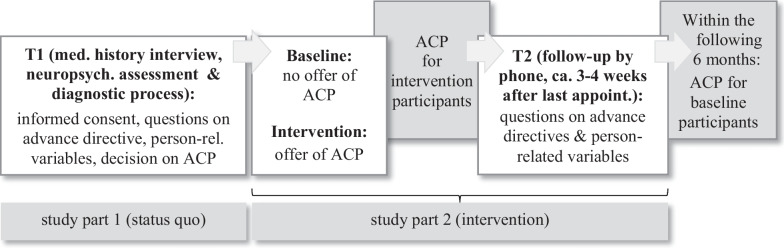
Procedure

The procedure is presented in Fig. 1. Data for study part 1 (status quo) is assessed at T0 and T1, data for study part 2 is assessed at T2.

In both memory clinics, participants with suspected dementia usually undergo three appointments: A medical history interview with the treating physician, a neuropsychological assessment, and an appointment for communicating the diagnosis. Moreover, the memory clinics try to follow-up on every patient, but this has not yet been performed reliably.

The study’s T1 incorporates the three appointments at the memory clinic. At the medical history interview (first appointment), participants consent to the present study (for details regarding informed consent, see below). Moreover, additional person-related variables not collected as part of the standard medical treatment will also be assessed, such as education, need for autonomy in medical decision making, health literacy, and comorbidities.

During the neuropsychological assessment (second appointment), the MMSE score is determined as a marker of cognitive decline and the score of the GDS [9] as a marker of depressive symptoms.

After communication of the diagnosis (third appointment), eligible participants in the intervention phase^1^ are asked whether they want to participate in ACP with the aim of creating an advance directive. If they consent, the study physician will arrange additional appointments besides the usual treatment to perform the ACP.^2^ The number and duration of these vary according to need. If not, a short questionnaire is filled out on the patient’s reasons for rejection.

Participants in the baseline phase^3^ do not receive the offer of ACP and are only contacted at follow-up (T2). For ethical reasons, they will be offered participation in ACP within the next 6 months after their appointments at the clinic (outside the present study).

As a consequence of this study, routine follow-up appointments via phone calls will be established as part of the memory clinic treatment. The primary aim of these follow-ups is to check if patients adhere to their medication or non-medical treatment and are able to handle any upcoming side effects. Moreover, questions from the patients or their caregivers about the diagnosis can be answered through these follow-up calls. For reasons of standardization, these follow-ups (T2) happen three to four weeks after a patient’s last appointment at the clinic.

The T2 questionnaire of this study records participation, outcome, and, if applicable, reasons for rejection, cancellation, or discontinuation of ACP (see Table 1). Participants will again be handed the advance directive questionnaire (see above). Participants who have not completed the ACP process at the time of the follow-up assessment will refer to a (possible) pre-existing advance directive (see statistical evaluation regarding sensitivity analyses on this topic). In addition, demographic data and the severity of dementia will be recorded. The study physician fills in all the answers known to him from the patient file or the ACP process to minimise the burden on the participants. If participants are not available on the phone at the agreed-upon time, the study physician will call them two more times before considering them lost to T2.

**Table 1:** Participation in ACP yes/no, reasons and consequences

Participation in ACP	Reason/result
No,	… because he/she was not offered ACP (*baseline phase*)
	… because he/she declined the offer of ACP
Yes,	… the full ACP process has been performed
	… but the ACP process has not been completed yet
	… but the he/she has cancelled/discontinued the process
	… but it turned out during the ACP process that the patient was unable to consent
Other	(please describe)

#### Non-adherence, retention, and withdrawal

Participants in the intervention phase who reject ACP are a vital part of the analysis since the *offer* of ACP is the intervention. Therefore, they remain part of the intervention group, and their decision to reject ACP will be recorded. The same applies to participants in the intervention phase who initially accept ACP but fail to attend all or part of the ACP appointments. Both groups are asked to fill in a short questionnaire on the reasons for rejection or cancellation (see section “Instruments”).

Of course, any participants may withdraw from the study at any time without giving reasons. Nonetheless, in order to avoid bias from selective withdrawal, we seek to increase retention by designing the study follow-up as a low-threshold telephone interview integrated into routine care, so participants will not need to be at the clinic for it and still have a benefit from the possibility to ask questions concerning their diagnosis and therapy.

### Instruments

All questionnaires are available to the interested reader on request. A decision flow chart for questionnaire presentation can be found in “Appendix A”.

#### Primary outcome advance directive: existence and validity

Information on the existence and validity of the advance directive is collected at T0 in a self-report questionnaire and at T2 in a telephone interview. The closed questions were specifically composed for this study.

First, the existence of an advance directive is recorded (“Do you have an advance directive? Yes/no). This question acts as a filter: If no advance directive is available, none of the subsequent questions follows. If the answer is yes, the validity of the advance directive is assessed.

Since advance directives are highly-sensitive personal data, determining their validity via their content is ethically problematic in our view. One approach, therefore, is to ask whether informed consent was given by the participant when the declaration was drawn up, meaning (1) a patient capable of giving consent, (2) had received adequate information to make an informed decision, and (3) this decision had been voluntary [10]. The respective questions with dichotomous response options (yes/no, supplemented by “don’t know”), can be found in Table 2.

**Table 2:** Questions on the validity of the advance directive

	Question
Prerequisite	Has the advance directive been made with a notary, physician or psychotherapist?
Adequate information	Has there been a medical/psychotherapeutic consultation?
	Has there been a notarial consultation?
Capacity to consent	Has your capacity to consent been documented by the physician/psychotherapist or the notary when the advance directive was drawn up?
Voluntariness	Did you create the advance directive voluntarily?

Besides asking about participants’ advance directive, in this questionnaire, we also ask about the existence of a guardianship, a guardianship directive, or a lasting power of attorney.

#### Secondary outcome advance directive: satisfaction

Satisfaction with the (latest) advance directive is assessed at T0 and T2.

In order to minimize time expenditure, a single item with a five-point response scale is used to record satisfaction with the respective advance directive ("How much do you agree to the following sentence: I am satisfied with my advance directive.—Does not apply at all—Applies very much"; also see Lattuca et al., 2018), supplemented by a “don’t know” option.

#### Rejection of ACP

For participants in the intervention group who decline the ACP offer, a closed-ended question will ask about reasons for refusal. The response alternatives are presented in Table 3.

**Table 3:** Reasons for rejecting ACP

*Please circle the answer that applies to you. Multiple answers are possible* *I don’t want to take part in ACP because…*	
• I don’t want to create my advance directive • I already have an advance directive that I am satisfied with • The whole process is physically/mentally too demanding for me • The whole process is emotionally too demanding for me • I prefer to make my advance directive later • I cannot make the additional appointments • Others (please name the reasons)	

#### Potential predictors of the existence of valid advance directives

In addition to demographic data (age, gender, education), the need for autonomy in medical decision making, health literacy, somatic and psychiatric comorbidities, as well as the severity of dementia are assessed.

Demographic data are collected at T1 and T2.

The need for autonomy in medical decisions will be assessed at T1 using a single item by [11], translated to German and adapted for the needs of this study. The item asks who should decide about a medical treatment; the answers range from *no need for autonomy* (decision is made solely by the physician) to a *very high need for autonomy* (decision is made solely by the patient) on a five-point scale.

Health literacy will also be assessed at T1 using a single item by [12], translated for this study. Participants are asked to rate, on a five-point scale, how much difficulty they have with reading medical information.

Comorbidities will be recorded at T1 with the help of the CIRS-G (Cumulative Illness Rating Scale-Geriatric) [13]. Using this questionnaire, the treating physician rates the severity of the damage to 13 body systems, functions, and organs, as well as the severity of psychiatric impairment. Severity ratings range from *no damage* to *severe damage* on a five-point scale. For the somatic damage, a sum score of the 13 single body damage ratings is calculated with the somatic morbidity index (SMI). The psychiatric impairment score is used as a single item.

In addition, the German version of the 15-item short form of the GDS [9] is used. It is a renowned screening instrument for depression in geriatric settings, which considers the differing symptoms depression may present in older age.

Finally, the MMSE, a well-known screening instrument is used at T1 and T2 to assess the degree of patients’ cognitive dysfunction. In this study, we use the MMSE version contained in the CERAD-Plus (neuropsychological test battery for dementia by the Memory Clinic Basel, see, e.g. [14]). It is routinely assessed by the treating neuropsychologist^4^ during the neuropsychological assessment at T1 and transferred to the T2 documentation. At T2, when the final diagnosis has been made, the severity of dementia is recorded by a single item reflecting the diagnosis communicated to the patient (no dementia—mild cognitive impairment—mild dementia—mild to moderate dementia—moderate dementia—severe dementia^5^—not yet diagnosed).

### Statistical evaluation

#### Statistical methods of evaluation

The first two research questions of study part 1 (about the presence and validity of advance directives and the patients’ satisfaction with them) are merely descriptive. For research question 3 (about the association between patient-related characteristics and the presence of a (valid) advance directive), a logistic regression model is fitted with and without consideration of the validity of the advance directive, respectively. Factors entered into the model are demographic variables, need for autonomy in medical decision-making, health literacy, comorbidity, and the severity of dementia as assessed by MMSE. All data used in these analyses are gathered at T1.

In study part 2 (intervention), question 4 (the hypotheses that the proportions of patients with either only a valid advance directive or with an advance directive, regardless of its validity, are higher after the offer of ACP than during the baseline phase), will be tested using a chi-squared test for comparison of two independent samples. To allow for cases of incomplete ACP at the time of assessment (T2), a sensitivity analysis that considers the potential of valid advance directives will be conducted.

Confirmatory testing is only planned for the first hypothesis in question 4 (that the weekly proportions of patients with a *valid* advance directive are higher during the intervention phase). So alpha error adjustment for multiplicity is not necessary.

For question 5 (the association between ACP and satisfaction with advance directive), an asymptotic Wilcoxon-Mann–Whitney test for comparison of two independent samples will be used.

#### Missing data

Missingness of data might be a concern in research question 3 of study part 1 (the association between person-related variables and existence and validity of advance directives) and in study part 2 (comparison of the frequency of advance directives, valid advance directives, and of the patients’ satisfaction with their existing advance directives during the baseline vs. the intervention phase). In study part 2 (intervention study), we assume that most missing data is created by participants accidentally skipping a question. Therefore, the study person receiving the questionnaires from participants goes through them and asks participants to fill in any missing data. In order to avoid a loss to follow-up, we designed the study follow-up as a low-threshold telephone interview with the additional motivating element that patients can use as a means to contact their treating physician to ask questions concerning their therapy. Therefore, we assume that all missing data will be lost completely at random in the intervention study and that the amount of missing data will be very low so we can obtain unbiased test results from a complete case analysis. In study part 1, however, more missings can be expected because a wider range of participants takes part in the study, particularly those with pronounced deficits that impair answering the questionnaire items. To ensure the best possible data quality, information may be provided by relatives or proxies (e.g., the existence and validity of advance directive). However, this does not apply to questions regarding the participant’s personal opinion (e.g., satisfaction with the advance directive, health literacy, autonomy in medical decision-making). Assuming that cognitive impairment is primarily responsible for the missingness of these data, we include the MMSE score in our model as a mediator variable. Therefore, we expect to obtain unbiased regressors even if the data are only missing at random and not missing completely at random.

### Quality control

#### Data management

The Data Management Committee (DMC) is composed of a biostatician and a data manager, both not involved in data collection.^6^ It gives statistical advice, is responsible for data digitalisation, data quality assurance and privacy protection, and surveys the database. All the data will be recorded paper-based. Data relevant for patient treatment will be added to the electronic patient file. The online survey tool LimeSurvey will be used as a database.

The DMC is also responsible for data monitoring, focusing on aspects like missing or implausible values, and value distribution, thereby ensuring data quality. Double data entry is performed on one randomly selected record out of each set of ten successive records by a second person, using algorithms to assess the quality of the entered data. Detected discrepancies will lead to repeated data entry of each of the 10 records. After successful completion of the data entry process, paper-based records will be destroyed.

Since participants are exposed to only very minor risks during the study (see below), no interim analyses during the data assessment phase are planned.

#### Trial monitoring

A Trial Management Committee (TMC) elaborates the details of the study design and procedure and issues the data assessment and the informed consent materials. It meets on a weekly basis to monitor the progress of the study, initiate the next steps, report problems, and agree on solutions. Part of the TMC is a Steering Committee (SC) that oversees the whole trial. It reviews the progress of the study together with the DMC and, if necessary, agrees on changes to the protocol to facilitate the smooth running of the study. The SC keeps the study protocol and the entry to the study registry up to date and, if necessary, communicates amendments to all study members and the ethical review boards (as soon as possible), cooperating partners (quarterly), and the patient advisory board (see below; at its subsequent meeting). It also organizes meetings of the TMC.

#### Principal and lead investigators

The principal investigator designed the study and acquired the grant. She is responsible for the trial initiation and management (head of SC) and takes all the final decisions. It is mainly her who communicates with the associated partners.

Lead investigators are the respective study physician and neuropsychologist at each of the study centres, two leading investigators per centre. They are part of the TMC and form the link between researchers at the University and practitioners in the memory clinics by instructing and continuously informing the other investigators. Moreover, they enrol the patients and collect the study data. Finally, they are responsible for reporting any adverse events as reported by the participants or as observed by themselves back to the TMC.

Principal and lead investigators declare to have no competing interests.

#### Audits

We have installed an ethical advisory board for this study, not related to the University of Siegen as study sponsor, nor to any of our cooperating partners. Members of the Institute for Medical Ethics and History of Medicine at the Ruhr-University Bochum, Germany, will advise us on questions related to research ethics in bi-annual meetings, particularly regarding the problem of involving non-competent persons in research.

Scientific advice will come from international experts of different disciplines. We plan to conduct bi-annual expert workshops on research ethics, assessment of capacity to complete advance directives, and decision-making and task complexity, amongst others. Moreover, the theoretical input on the gerontological concepts of ageing-well will come from the cooperating Frankfurt Forum of Interdisciplinary Ageing Research.

Finally, we have established a patient advisory board at our Institute^7^ that will also be involved in the current study. The general aim of this advisory board is to inform and counsel researchers on practical and ethical questions arising during the research process. This will also allow our research to be closely adapted to the needs of PwAD and their caregivers. Presently, the patient advisory board consists of four patients who meet on a monthly basis. The participating patients receive written information about the next session’s topics beforehand if available. A member of the research group will present the current state of the study and asks the researchers’ questions for the members of the board to discuss. In the current study, besides considering ethical topics when they arise, questions will centre around ACP and expectations regarding its facilitation. This is aimed at gaining a deeper and broader understanding of the needs of PwAD with respect to ACP in preparation for a subsequent study.

### Ethics and dissemination

The study procedure and materials have been reviewed and approved by the Ethics Committee of the Medical Council Westfalen-Lippe and the Medical Faculty of the Westfälischen Wilhelms-Universität Münster, Germany, (trial no. 2021-518-f-S) and the Ethical Committee of the Medical Faculty at the Goethe University Frankfurt am Main (trial no. 2021-559). The study has been registered with the Germany Registry for Clinical Trials (DRKS, Deutsches Register Klinischer Studien, no. DRKS00026691). For interested readers, all items of the WHO Trial Registration Data Set can be found in “Appendix B”.

Any relevant amendments to this protocol will be communicated formally to both ethical committees for approval and to the study registry.

#### Possible harms, ancillary, and post-trial care

Apart from the minimal physical, cognitive, and emotional burden put onto participants by filling in the questionnaires, no harm is anticipated. Nonetheless, participants are encouraged to report any adverse events to the investigator collecting the data. Investigators are obliged to report any adverse event or unexpected effect reported to them or observed by them to the TMC (including the SC), which will decide on how to react. If ethically complex, members of the TMC may decide to present the problem to the patient and/or the ethical advisory board. Based on an evaluation of the potential concern, we will adapt our procedure within one months after the event. The adverse events and unexpected side-effects are also collected in the LimeSurvey data base for trial monitoring.

Post-trial care will be provided as part of the routine treatment at the memory clinic. We do not expect any need for ancillary care.

#### Informed consent and assent

At the first appointment, participants are informed about the study by the study physician and can discuss any questions or other issues they may have. The physician then obtains written consent if participants agree to take part. Participant information and informed consent form are available on request.

Since participants are suspected of dementia, the capability to consent could be an issue. To support the ability to consent, measures to increase the ability of people with dementia to consent to medical interventions according to the German AWMF S2k guidelines to medical procedures [10] will be used. Patients who cannot consent with the help of these supporting measures are excluded from the study as long as no proxy is available to consent in his/her stead. Nonetheless, consent by proxy still requires each patient’s assent. Visible or audible lack of assent at any point of the study will result in the participant’s withdrawal.

#### Confidentiality and access to data

After the assessment, paper-based records are locked up at the site of assessment (i.e., either of the memory clinics) until data entry into the database. Data relevant for patient treatment is also stored in each patient’s file. After data entry and quality control procedures have been performed, the records are destroyed. Since data is anonymized at the source, it cannot be related to the participants’ names at any point during the study.

Regarding researchers of the DECIDE project, only members of the DMC have access to the complete digitalized data set. Other than that, those graduating with project data will have access only to the data needed for their thesis. Regarding data access for researchers outside the project, the (anonymous) data on which the results of publications are based will be made available to other researchers outside the project via PsychData, a German data-sharing platform for psychological research (www.psychdata.de). Study protocols and codebooks are also going to be shared. The data will be made available between six months and ten years after publication to researchers who put forward reasonable requests for data usage and are willing to sign the data usage agreement. The data may only be used for the analyses specified in the request.

#### Dissemination of results

The scientific findings will preferably be published in open-access journals which employ recognised, strict quality assurance processes (i.e., peer reviewed journals). Every listed author will have made a substantial, direct, intellectual contribution to the manuscript. We do not intend to use any professional writers.

Moreover, it is planned that the findings will be integrated into the updated version of the AWMF-Guideline “Einwilligung von Menschen mit Demenz in medizinische Maßnahmen” (consent to medical treatment by people with dementia). According to the AWMF manual for guideline development, members of the guideline group should have professional and scientific expertise as well as patient experience, which means that both researchers and laypersons will be involved.

Finally, the study results will also be presented at both national and international conferences on dementia and geriatrics.

## Discussion

The current study treats two main topics. Firstly, it investigates the status quo of (valid) advance directives in the population of memory clinic patients, since no reliable data is available on the proportion of PwAD with valid advance directives in Germany and worldwide. The results are highly relevant regarding whether action should be taken to increase the amount of valid and satisfying advance directives in this population. If found that the proportion of PwAD with valid advance directives is low, measures need to be put in place to assure the implementation of Article 12 of the UN Convention on the Rights of Persons with Disabilities, thus enabling PwAD to exercise their right to self-determined medical decision making.

In addition, we aim at exploring the predictors of the existence of valid advance directives since to the best of our knowledge no data exists regarding this question. Associations between variables like, e.g., demographics, literacy in and attitudes towards health-related decisions, with or without a (valid) advance directive can help to identify populations at risk of deprivation of their rights for autonomy and develop needs-based enhanced consent procedures. Groups at risk would be the first targets for interventions, which could be tailored to their needs.

We could find that the proportion of valid advance directives is very high in the target population, independent of any person-related factors and that there is no need for further intervention. However, this result is highly unlikely since this would require the proportion of advance directives to be higher in the population of PwAD than in the general population. Rather, we expect the opposite and a high need for interventions in this regard.

Therefore, in the second part of this study, we investigate whether participants benefit from the offer of ACP, a particular process for supporting laypersons in creating valid advance directives together with experts like physicians, psychotherapists, and notaries. Although ACP itself is not part of the intervention, this study is one of the first to target the possibility of interventions aimed at increasing the number of valid advance directives among PwAD. We assume that the proportion of valid advance directives will increase by *offering* ACP to participants because it may trigger the willingness of PwAD to (re)think advance directives and create one with or even without the use of ACP.

Some possible pitfalls of this study have to be outlined. Firstly, PwAD can be difficult to enroll in studies. However, we have taken care to design the threshold for study access as low as possible: We recruit from a convenience sample of memory clinic patients, i.e., participants only need to show up for additional appointments if they want to take part in ACP. Also, the burden put on participants by the study is minimal since it requires only about 15 min of filling in questionnaires, as most of the data assessed are part of routine care. Participants do not have to present themselves at the clinic for follow-up, instead a phone interview is performed to gather data for the second assessment, which will increase the likelihood to retain participants in the study.

Nonetheless, the sample size is, of course, limited by the number of patients presenting at the memory clinic. To reach a satisfactory sample size, we need the physicians to see at least five patients each week in each of the participating clinics. This reasonably small number should not be an issue to either of the two clinics since, in the general population, demand for diagnosis and treatment of memory problems in older age is high. In addition, the funding allows us to support the two sites with extra man-power for medical consultation and neuropsychological assessment.

Finally, we do not analyse the content of the patients’ existing advance directives, which would add information on their validity. Instead, our focus is on the formal criteria of the presence or absence of informed consent. We decided against the analysis of the content of advance directives for two main reasons: Firstly, such analysis would require a high number of person-hours by raters highly skilled in determining the validity of advance directives content. Secondly and more importantly, advance directives are highly private, and the information contained is very personal. The threshold for study participation would rise, and we could not keep the data as anonymous as we do now. Therefore, analysing the content of memory clinic patients’ advance directives is an objective open to future studies.

Future studies in the project DECIDE will be based on the results of the present study. It aims at increasing the number of valid advance directives in PwAD by a spatial intervention based on the assumption that the clinical setting is suboptimal for PwAD to make treatment decisions. As a result of this study, specific subgroups of the population of PwAD may then be given particular attention, either because they lack advance directives to a larger extent or because they are particularly prone to denying ACP in the clinical setting.

All in all, the present study will be the first to shed light on the benefit of offering ACP to PwAD and will entail further studies in this emerging field of research. The complete project DECIDE is designed to increase the knowledge on how to assist PwAD to exercise their right to self-determined treatment decisions since there is a dearth of articles on how to effectively utilise Article 12 of the UN Convention on the Rights of People with Disabilities for PwAD.

## Figures and Tables

**Figure:**
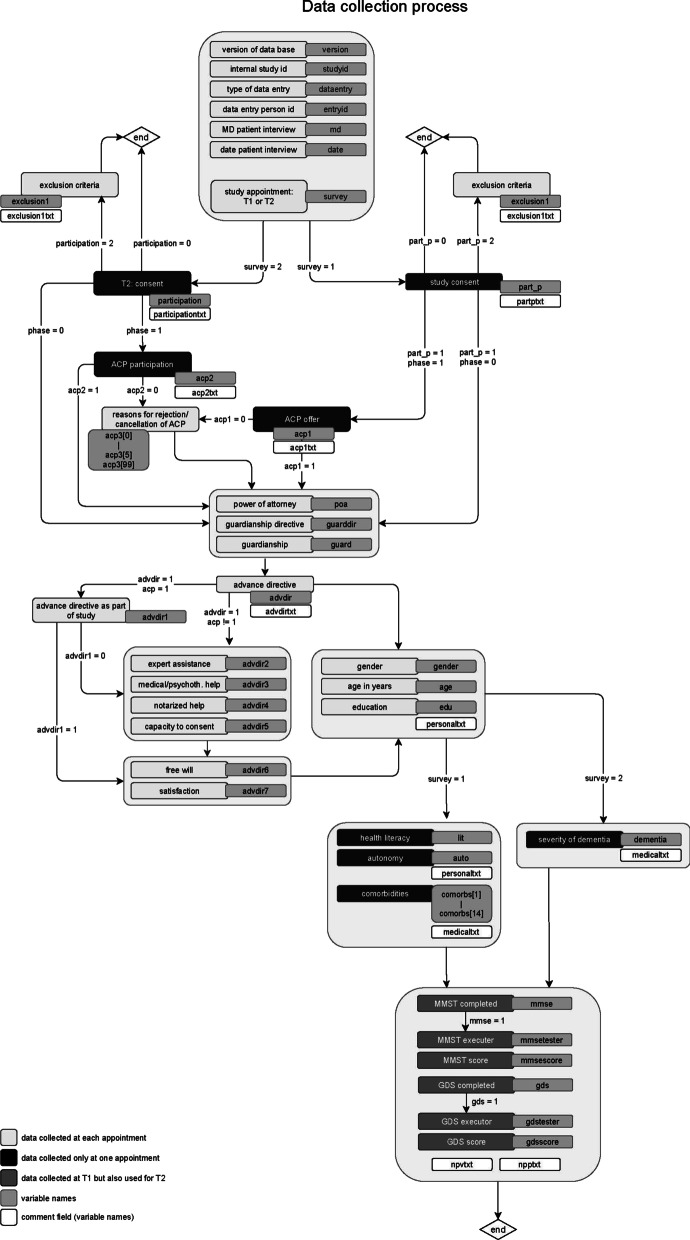
Decision flow chart for questionnaire presentation

## Data Availability

The datasets generated and analyzed during the current study are available in the PsychData repository, www.psychdata.de (permanent link not available at time of publication due to ongoing data assessment) on reasonable request.

## Appendix B: WHO trial data registration set

**Table:**

Category	Information
Primary registry and trial identifying number	drks.de DRKS00026691
Date of registration in primary registry	15th of October 2021
Source(s) of monetary or material support	German Federal Ministry of Education and Research
Primary sponsor	Universität Siegen
Secondary sponsor(s)	–
Contact for public queries	Julia Haberstroh, Universität Siegen, 0049 (0)271 740-4053, julia.haberstroh@uni-siegen.de
Contact for scientific queries	Julia Haberstroh, Universität Siegen, 0049 (0)271 740-4053, julia.haberstroh@uni-siegen.de
Public title	Project DECIDE part I: Increasing the amount of valid advance directives in people with Alzheimer’s Disease
Scientific title	Project DECIDE – process intervention: Increasing the amount of valid advance directives in people with Alzheimer’s Disease by offering advance care planning
Countries of recruitment	Germany
Health condition(s) or problem(s) studied	Lack of valid advance directives in people with Alzheimer’s Disease
Intervention(s)	Intervention: offer of advance care planning No intervention: no offer of advance care planning
Key inclusion and exclusion criteria	Inclusion criteria: Suspected mild to moderate dementia Exclusion criteria: Severe dementia Delirium Intellectual disability Severe mental illness Lack of capacity to consent to medical research with simultaneous incapacity for supported decision making by a relative or proxy Uncompensated pronounced sensory deficits Insufficient knowledge of the German language which makes the understanding of the study documents and/or the interview impossible
Study type	Interventional Allocation: blockwise Intervention model: parallel assignment Masking: none Primary purpose: enhancement of patient autonomy
Date of first enrolment	November 2021
Target sample size	250
Recruitment status	Recruiting
Primary outcome(s)	Advance directives, validity of advance directives
Key secondary outcomes	Satisfaction with advance directive

## Abbreviations

PwAD: People with Alzheimer’s disease
ACP: Advance care planning

## Footnotes

1 Those eligible for study part 2 (see exclusion criteria of study part 2). Reasons for exclusion are recorded.

2 ACP will be performed following the Respecting Choices Approach®. In each of the two clinics, one member of staff (Siegen: a doctor, Frankfurt: a psychotherapist) will go through the process with the participants in as many sessions as necessary. These experts (“Gesprächsbegleiter”) are trained by the Würdezentrum Frankfurt (for more details, see https://www.wuerdezentrum.de/wp-content/uploads/2019/12/BVP-Flyer_2019_10_09_15_20_51.pdf).

3 For ethical reasons, ACP will also be offered to baseline participants but only after their follow-up appointment (T2) as opposed to intervention participants who receive the offer at T1.

4 Unless no neuropsychological assessment can take place, in which case the study physician will administer the MMSE.

5 Patients with severe dementia at T2 are excluded at T2.

6 Both are employed by the study sponsor, which has no role in study conception, data assessment, and evaluation or any other aspects of the study.

7 In cooperation with the Alzheimer Gesellschaft Siegen (Alzheimer Society Siegen).
